# Differential Regulation of Echinocandin Targets Fks1 and Fks2 in *Candida glabrata* by the Post-Transcriptional Regulator Ssd1

**DOI:** 10.3390/jof6030143

**Published:** 2020-08-21

**Authors:** Kelley R. Healey, Padmaja Paderu, Xin Hou, Cristina Jimenez Ortigosa, Nicole Bagley, Biren Patel, Yanan Zhao, David S. Perlin

**Affiliations:** 1 Department of Biology, William Paterson University, 300 Pompton Road, Wayne, NJ 07470, USA; bagleyn@student.wpunj.edu (N.B.); patelb33@student.wpunj.edu (B.P.); 2 Center for Discovery and Innovation, Hackensack Meridian Health, 111 Ideation Way, Nutley, NJ 07110, USA; Padmaja.Paderu@hmh-cdi.org (P.P.); houxin@pumch.cn (X.H.); cristina.jimenez-ortigosa@hmh-cdi.org (C.J.O.); Yanan.Zhao@hmh-cdi.org (Y.Z.); David.Perlin@hmh-cdi.org (D.S.P.); 3 Department of Clinical Laboratory, Beijing Key Laboratory for Mechanisms Research and Precision Diagnosis of Invasive Fungal Diseases (BZ0447), Peking Union Medical College Hospital, Chinese Academy of Medical Sciences, Beijing 100730, China; 4 Department of Medical Sciences, Hackensack Meridian School of Medicine, 340 Kingsland Street, Nutley, NJ 07110, USA

**Keywords:** *Candida glabrata*, echinocandin resistance, FKS mutation, FKS regulation, SSD1

## Abstract

Invasive infections caused by the opportunistic pathogen *Candida glabrata* are treated with echinocandin antifungals that target β-1,3-glucan synthase, an enzyme critical for fungal cell wall biosynthesis. Echinocandin resistance develops upon mutation of genes (*FKS1* or *FKS2*) that encode the glucan synthase catalytic subunits. We have analyzed cellular factors that influence echinocandin susceptibility and here describe effects of the post-transcriptional regulator Ssd1, which in *S. cerevisiae*, can bind cell wall related gene transcripts. The *SSD1* homolog in *C. glabrata* was disrupted in isogenic wild type and equivalent *FKS1* and *FKS2* mutant strains that demonstrate echinocandin resistance (MICs > 0.5 µg/mL). A reversal of resistance (8- to 128-fold decrease in MICs) was observed in *FKS1* mutants, but not in *FKS2* mutants, following *SSD1* deletion. Additionally, this phenotype was complemented upon expression of *SSD1* from plasmid (p*SSD1*). All *SSD1* disruptants displayed susceptibility to the calcineurin inhibitor FK506, similar to *fks1∆*. Decreases in relative gene expression ratios of *FKS1* to *FKS2* (2.6- to 4.5-fold) and in protein ratios of Fks1 to Fks2 (2.7- and 8.4-fold) were observed in *FKS* mutants upon *SSD1* disruption. Additionally, a complementary increase in protein ratio was observed in the p*SSD1* expressing strain. Overall, we describe a cellular factor that influences Fks1-specific mediated resistance and demonstrates further differential regulation of *FKS1* and *FKS2* in *C. glabrata*.

## 1. Introduction

Infections caused by *Candida glabrata*, especially among immunocompromised hosts, have increased in prevalence and demonstrate elevated rates of antifungal resistance [1,2,3]. The echinocandins (caspofungin, micafungin, and anidulafungin) are recommended first-line agents for the treatment of invasive *C. glabrata* infections [4]. Echinocandin antifungals target the plasma membrane-embedded enzyme β-1,3-glucan synthase, leading to loss of β-glucans and cell wall stability. Resistance to the echinocandins is well-established (3–12%) among patients with *C. glabrata* infections and develops upon mutation of genes (*FKS1* or *FKS2*) that encode for the catalytic subunits (Fks1/Fks2) of β-glucan synthase [1,2,5].

Although fungi contain multiple *FKS* paralogs (i.e., *FKS1*, *FKS2*, and *FKS3*), *FKS1* is an essential gene in most, including *Candida* and *Aspergillus* species. Notable exceptions are *Saccharomyces cerevisiae* and *C. glabrata*, where *FKS1* and *FKS2* are considered functionally redundant. In these organisms, any one *FKS* gene can be disrupted and cell growth can continue; however, *FKS1* and *FKS2* cannot be simultaneously disrupted [6,7].

In *S. cerevisiae*, *FKS1* expression is regulated in the cell cycle and predominates during growth on glucose, while *FKS2* (also referred to as *GSC2*) has been shown to be important during sporulation, growth in the absence of glucose, cell wall stress, and osmotic shock [6,8]. Expression of *FKS2* in *S. cerevisiae* is induced by the cell wall integrity pathway [9], which is activated under various stressors, and by the calcium/calmodulin-dependent protein phosphatase calcineurin [6]. Interestingly, sporulation and mating have not been observed in *C. glabrata*, despite the presence of mating genes in its genome [10,11,12]. Additionally, while *FKS1* in *S. cerevisiae* is predominantly expressed unless one of the above stressful conditions arises, *FKS2* in *C. glabrata* is expressed more broadly at levels comparable to *FKS1* [13,14,15]. The apparent increased role of *FKS2* in *C. glabrata* is the likely reason that mutations within this gene can lead to echinocandin resistance. In fact, mutations in either *FKS1* or *FKS2* are routinely identified within resistant clinical isolates of *C. glabrata*, while other medically-relevant fungi exclusively develop *FKS1* mutations [5].

Because mutations in either *FKS1* or *FKS2* in *C. glabrata* can lead to echinocandin resistance and treatment failure, it is important to understand how this yeast regulates these drug targets and whether this regulation is common or unique to each target. Compared to *S. cerevisiae*, the genetic regulation of *FKS1* and *FKS2* expression in *C. glabrata* has been less studied. Large scale transcriptomic studies have reported changes in *C. glabrata FKS1* or *FKS2* expression under specific conditions. *FKS1* was upregulated during growth in a low pH medium following disruption of the pH response regulator *ASG1* [16], and *FKS2* was upregulated following acetic acid treatment of wild type or *HAA1*-deleted cells [17]. In each study, the opposing *FKS* gene was not reported as exhibiting a change in expression. While it has been shown that resistance mutations in *FKS* genes can alter downstream expression [14], only one mechanism of differential regulation linked to antifungal susceptibility, to our knowledge, has been characterized in *C. glabrata*: transcriptional regulation of *FKS2* by calcineurin. As in *S. cerevisiae* [6], chemical or genetic inhibition of calcineurin decreases expression of *FKS2*, but not *FKS1*, in *C. glabrata* [7]. As such, a partial reversal in echinocandin resistance was observed in *C. glabrata FKS2* mutants, but not *FKS1* mutants, following treatment with the calcineurin inhibitor FK506 (tacrolimus) [7].

Ssd1 is an RNA-binding protein that promotes or represses translation. In *S. cerevisiae*, Ssd1 shuttles into and out of the nucleus and binds cell wall mRNA transcripts, including that of chitinases and glucanases [18,19]. A Cbk1-phosphorylated Ssd1 will deliver its bound mRNA to the proper site for translation, while a dephosphorylated Ssd1 will sequester the bound mRNA and prevent its proper localization and translation [20,21]. Overall, Ssd1 is proposed to directly modulate the delivery, retention, and translation of mRNAs [21], thereby regulating cell wall growth and remodeling.

Here, we show that Ssd1 is differentially regulating the catalytic subunits (Fks1 and Fks2) of β-1,3-glucan synthase in *C. glabrata*. Upon *SSD1* deletion within *FKS* mutant backgrounds, we observed a reversal of Fks1-mediated resistance, FK506 sensitivity, decreased *FKS1* gene and protein expression, and a complementary increase in *FKS2* gene and protein expression, suggesting regulation, either directly or indirectly, of *FKS1* by Ssd1.

## 2. Materials and Methods

### 2.1. Strain Construction and Media

*Candida glabrata* 2001 *(*CBS138*)* and 200989 (2001 *his-, trp-, ura-)* strains were obtained from American Type Culture Collection (ATCC, Manassas, VA, USA). The *FKS1* and *FKS2* gene knockouts in ATCC 200989 were gifts from S. Katiyar (Drexel University College of Medicine) [7]. Fks1 (625delF and S629P) and Fks2 (659delF and S663P) mutants were generated in strain ATCC 200989 through transformation of a purified PCR product. Specific mutations were PCR-amplified along with regions flanking the *FKS1* or *FKS2* hotspot 1 region (approximately 400 bp) from mutant isolates (see Table S1 for primers). Transformants were selected on low levels (0.2 µg/mL) of caspofungin-containing YPD (1% yeast extract, 2% peptone, 2% dextrose) agar medium. All *FKS1* and *FKS2* hotspots were sequenced in the transformants to confirm the expected mutation was present and all other amino acids remained unchanged.

To disrupt *SSD1*, *S. cerevisiae TRP1* was amplified from pRS414 with primers that contained overhangs homologous to the up- and down-stream regions of *C. glabrata SSD1* (Table S1). This deletion cassette was purified and transformed into competent wild type cells. Transformants were selected on complete defined agar medium without tryptophan (SD-trp) (Sunrise Science Products, San Diego, CA, USA) and PCR screened for correct integration of *TRP1* at the *SSD1* locus and loss of *SSD1* (primers in Table S1). The ∆ssd1::Sc*TRP1* cassette was subsequently amplified and purified from this parental knockout providing longer regions of homology in order to transform each *FKS* mutant strain. All transformants were screened as noted above.

Plasmid pCN-PDC1 [22], which contains a strong promoter (*PDC1*), was used to constitutively express *SSD1*. A gap-repair approach [23] was used to clone *SSD1* into this plasmid. Briefly, the coding region of *SSD1* was PCR amplified from ATCC 2001 genomic DNA with primers that contained overhangs homologous to each side of the EcoRV restriction site (Table S1) ensuring proper directionality. The purified PCR product was co-transformed with EcoRV-linearized and alkaline phosphatase-treated pCN-PDC1 into competent yeast cells. Following the transformation, cells were subjected to a 3 h outgrowth in YPD broth followed by selection on YPD agar medium supplemented with 100 µg/mL nourseothricin (Jena Bioscience, Jena, Germany). Transformants were PCR screened for correct construct presence (Table S1). Plasmid DNA was rescued from yeast cells, purified, sequenced, and propagated in *E. coli* as in [24] and used to transform additional strains.

### 2.2. Drug Susceptibility Assays

Echinocandin minimum inhibitory concentrations (MICs) were determined by broth microdilution following CLSI standards [25]. Susceptibility assays were performed with YPD broth due to multiple, nutritional auxotrophies within the strains. Caspofungin (Merck, Rahway, NJ, USA) and micafungin (Astellas, Deerfield, IL, USA) were dissolved and diluted according to CLSI recommendations. The calcineurin inhibitor FK506/tacrolimus (Cayman Chemical, Ann Arbor, MI, USA) was dissolved in DMSO according to supplier recommendations.

The in vitro killing assays were performed as in [26]. Briefly, cultures of *C. glabrata* (1 × 10^7^ cells) in fresh 1 mL RPMI medium (plus necessary amino acids) were incubated at 37 °C while shaking for 24 h in 2-fold increasing concentrations (0.016 to 32 µg/mL) of echinocandin. After 24 h, 100 µL of the appropriate dilutions for each culture were plated onto YPD agar. Colony forming units (CFUs) were counted 24 h after plating. Two plates per concentration were analyzed and a minimum of two independent experiments were performed. Data are presented as CFU/mL that survived each drug concentration.

### 2.3. RNA Isolation and Quantitative RT-PCR

Cells were grown in YPD or YPD supplemented with 100 µg/mL nourseothricin (plasmid carrying strains) to mid-logarithmic phase. Total RNA was extracted using the RNeasy Mini kit (Qiagen Science, Germantown, MD, USA) according to the manufacturer’s instructions and stored at −80 °C. The concentration and purity of the RNA was determined using a UV spectrophotometer (NanoDrop One, Thermo Fisher Scientific, Waltham, MA, USA) by measuring the absorbance at 230 (OD230), 260 (OD260) and 280 nm (OD280). The integrity of the RNA was further checked by electrophoresis through 1% denaturing and non-denaturing agarose gels. *FKS1* and *FKS2* expression levels were measured by RT-PCR.

All qPCR reactions were performed in a 25-µL reaction mixture consisting of 12.5 µL of 2x One Step RT-PCR buffer (One Step SYBR Ex Taq qRT-PCR kit; TaKaRa Bio Inc., Mountain View, CA, USA), 0.2 µM of each primer, 0.5 µL Takara Ex Taq HS (5 U/µL), 0.5 µL RTase Enzyme Mix and 2 µL of RNA (5 ng/µL) on an Mx3005P real-time instrument (Stratagene, La Jolla, CA, USA). Optimal thermal cycling conditions consisted of 42 °C for 5 min for the reverse transcription, followed by an initial denaturation step at 95 °C for 10 s, 40 cycles of 95 °C for 5 s (denaturation), 60 °C for 20 s (annealing and extension). The experiments were carried out in triplicate for each data point. The relative quantification in gene expression was determined using the 2^−ΔΔCt^ method [27] with expression level of the gene *RDN5.8* for normalization [28]. The primers used are listed in Table S1. Statistical analysis was carried out using the Student’s *t* test (two-tailed) with SPSS software (version 12.0, SPSS Inc., Chicago, IL, USA), and *p* value of < 0.05 was considered significant.

### 2.4. Glucan Synthase Preparation and Western Blotting

Glucan synthase was isolated from strains as previously described [29]. Briefly, large volumes (2 L) of cells were harvested at early stationary phase, disrupted, and membranes were separated and isolated by sedimentation (100,000× *g*). Glucan synthase extraction and enrichment by product entrapment were performed as in [29]. Western blotting was performed as in [30]. Of note, proteins were prepared using Tris-Glycine-SDS buffer and reducing agent, heated, and separated by electrophoresis (8% Tris-Glycine gel). Following transfer of proteins to a PVDF membrane, blots were incubated with either anti-Fks1 or anti-Fks2 primary antibodies (GenScript Biotech, Piscataway, NJ, USA) at a dilution of 1:5000 or 1:3000, respectively, in 2% TBST overnight at 4 °C. Washed membranes were then incubated with horseradish peroxidase-conjugated secondary antibodies (anti-rabbit; Cell Signaling Technology, Boston, MA, USA) at 1:3000 dilution for 1 h. Bands were visualized with Novex ECL Chemiluminescent substrates (Thermo Fischer Scientific), and band intensities were determined with ImageJ software (https://imagej.nih.gov/ij/). Statistical analysis was carried out as stated above.

## 3. Results

### 3.1. SSD1 Disruption Reverses FKS1-Mediated Resistance in C. glabrata

**Figure 1:**
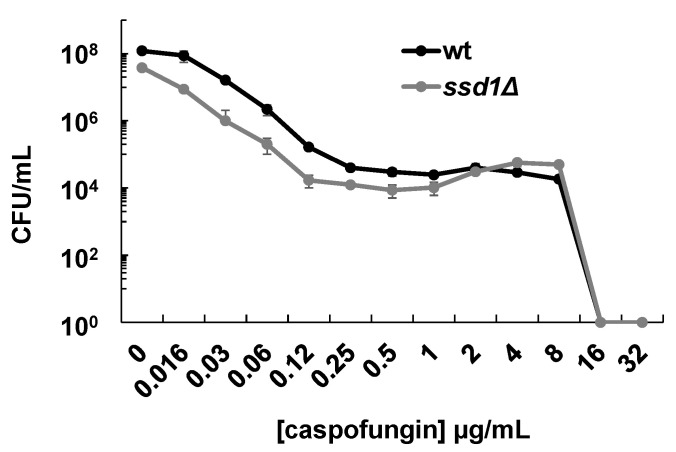
Disruption of predicted post-transcriptional regulator *SSD1* produces minor increases in echinocandin killing. In vitro caspofungin killing assay of wild type and *SSD1* knockout strains. Mean plus/minus SD of 2 independent experiments (each with 2 replicates) is shown. CFU, colony forming units.

Upon screening multiple gene disruptants in *C. glabrata*, we found that *ssd1∆* displayed 2- to 4-fold increases in susceptibility to caspofungin and micafungin as demonstrated by broth microdilution and killing assays (Table 1 and Figure 1). Disruption of *SSD1* in *C. glabrata* (CAGL0H01287g) was previously shown to elicit increased susceptibility to caspofungin [31]. The increases in echinocandin susceptibilities were similar to that of the *fks1∆* deletion strain (Table 1). In order to determine if the post-transcriptional regulator Ssd1 influences echinocandin resistance, we built clinically relevant *FKS1* and *FKS2* mutations into the ATCC 200989 background strain. The resulting equivalent amino acid alterations included Fks1-625delF, Fks2-659delF, Fks1-S629P, and Fks2-S663P. As expected, these *FKS1* and *FKS2* mutants demonstrated resistance with 32- to 64-fold increases in caspofungin and micafungin minimum inhibitory concentrations (MICs) compared to the non-mutated wild type strain (Table 1). We subsequently deleted *SSD1* in each mutant. While no significant changes in susceptibility were demonstrated by the *FKS2* mutants, we observed complete, or near complete, reversal of echinocandin resistance upon *SSD1* disruption in both *FKS1* mutants (Table 1). *SSD1* was then cloned onto a plasmid under control of a constitutive promoter and transformed into our mutant strains. Resistance was restored in each *FKS1 ∆ssd1* strain following expression of *SSD1* from the plasmid (Table 2). This complementation revealed that the presence or absence of *SSD1* was producing the observed changes in echinocandin susceptibilities and further raised the likelihood that *SSD1* was specifically modulating *FKS1* expression.

### 3.2. SSD1 Disruption Causes Increased Sensitivity to FK506

We used the calcineurin inhibitor, FK506 (tacrolimus), to determine the functionality of Fks1 in the *ssd1∆* strains. As *FKS2* expression is known to be dependent upon calcineurin signaling, the *fks1∆* strain demonstrates hyper-susceptibility (MIC ≤ 0.5 µg/mL) to FK506 (Table 1), as previously described [7]. Strains that can properly express Fks1 (e.g., wild type or *fks2∆*) are not hyper-susceptible to this inhibitor (MICs ≥ 32 µg/mL) (Table 1). We found that deletion of *SSD1* from the wild type strain or any *FKS* mutant caused increased susceptibility to FK506 (Table 1), consistent with the hypothesis that deletion of *SSD1* leads to a subsequent decrease in functional Fks1.

### 3.3. Loss of SSD1 Leads to Decreases in FKS1:FKS2 Gene and Fks1:Fks2 Protein Expression Ratios

**Figure 2:**
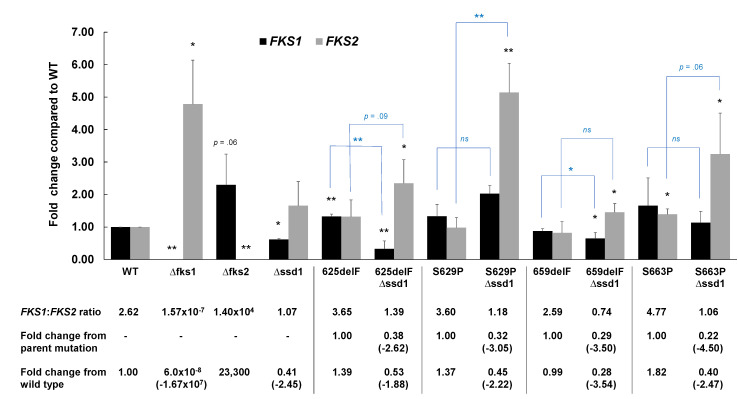
*SSD1* deletion leads to general decreases in *FKS1* gene expression and increases in *FKS2* gene expression. RNA was isolated from cells harvested at mid-log phase and expression compared to that of the wild type cells (2^−∆∆Ct^). Expression was normalized to *RDN5.8*. Mean plus SD of 3 independent experiments is shown. * *p* < 0.05, ** *p* < 0.01, *ns* = not significant; Student’s *t*-test, two-tailed. *p* values in black compare expression to wild type while brackets and *p* values in blue compare the same *FKS* mutant with and without *SSD1*. Relative expression ratios of *FKS1* to *FKS2* are displayed below the figure with associated fold changes from wild type or parental mutant.

To begin to understand how Ssd1 is regulating *FKS1* and/or *FKS2*, we first measured *FKS* gene expression in our strains. RNA was isolated from cells harvested in mid-log growth phase and levels of *FKS1* and *FKS2* mRNA were compared to that of the wild type strain. Relative expression ratios between *FKS1* and *FKS2* were also determined. As expected, control strains containing deletions of either *FKS1* or *FKS2* yielded significant increases in expression of the remaining *FKS* gene (Figure 2). The *SSD1* deletion strain demonstrated a significant decrease in *FKS1* expression and a non-significant increase in *FKS2* yielding a 2.45-fold decrease in *FKS1*:*FKS2* ratio compared to wild type (Figure 2). Nearly all *FKS1* and *FKS2* mutants demonstrated both decreases in *FKS1* expression and increases in *FKS2* expression upon *SSD1* disruption; some changes reached the level of significance (*p* < 0.05), while others did not (Figure 2). Consequently, decreases in *FKS1*:*FKS2* ratios were observed in each mutant deleted for *SSD1* when compared to either wild type (1.9- to 3.6-fold) or to the related parental strain (2.6- to 4.5-fold) (Figure 2). The increases in *FKS2* gene expression may be a direct result of *SSD1* deletion or a compensatory reaction of the cell to account for any loss in Fks1 protein, similar to the *fks1∆* strain.

**Figure 3:**
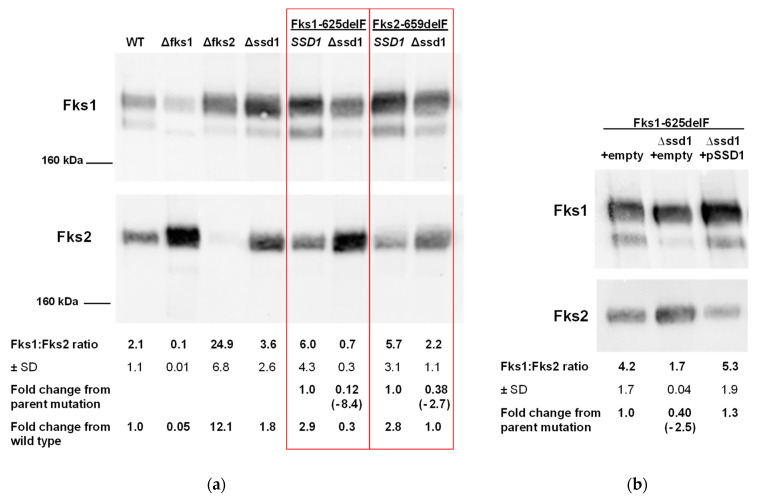
*SSD1* deletion leads to decreases in Fks1:Fks2 protein ratios in *FKS1* mutants. (**a**) *SSD1* deletion in the *FKS* mutant backgrounds (squared in red) leads to a decrease in Fks1:Fks2 protein ratio. (**b**) Plasmid expression of *SSD1* complements the decrease in Fks1:Fks2 protein ratio observed in the *fks1 ∆ssd1* mutant. Glucan synthase enzyme was purified from strains and western blotting performed with anti-CgFks1 or anti-CgFks2 antibodies that recognize N-terminal epitopes. ImageJ software was used to determine band intensities (integrated densities) and ratios of Fks1 to Fks2 were calculated. Representative blots from two independent experiments and mean ratios plus/minus SD are shown.

Next, we measured Fks1 and Fks2 protein levels within our strains. To do so, we isolated the glucan synthase enzyme from each strain and performed western blotting with anti-CgFks1 or anti-CgFks2 primary antibodies. Band intensities were determined with ImageJ software and data expressed in a ratio of Fks1 to Fks2 to normalize for enzyme quantities across strains. Mean decreases (2.7- and 8.4-fold) in the proportion of Fks1 to Fks2 were found in *FKS* mutant strains without *SSD1* compared to the same strains with the chromosomal copy of *SSD1* intact (Figure 3a). While the changes in Fks1:Fks2 protein ratios due to *FKS1* or *FKS2* disruption (control strains) reached the level of significance (*p* < 0.05), the decreases in Fks1:Fks2 within the mutant strains did not. However, these data are consistent with the aforementioned gene expression changes. As previously indicated [30], the anti-CgFks1 antibody may exhibit a small amount of cross-reactivity with Fks2, as evidenced by the faint Fks1 band from *fks1∆* cells; therefore, fold changes from wild type ratios are also displayed. The same analyses were also performed with several of the plasmid-carrying strains (Figure 3b). As in Figure 3a, a decrease in Fks1:Fks2 ratio was demonstrated upon *SSD1* disruption within the Fks1-625delF mutant. Importantly, upon complementation with p*SSD1*, the protein ratio returned to parental mutant levels (Figure 3b), indicative of a reversal in the protein changes observed with *SSD1* deletion.

## 4. Discussion

Invasive infections caused by *Candida glabrata* continue to exhibit increased rates of echinocandin resistance with acquired resistance occurring most prominently with either repeated or prolonged drug exposure or among patients with compromised immunity. This organism’s ability to swiftly and adeptly adapt to its environment in order to survive drug exposure is not fully understood. Resistance-conferring mutations, including those of *FKS1* and *FKS2*, are a final escape adaptation during prolonged drug exposure [32], and the presence of *FKS* mutations within patient samples is correlated with echinocandin treatment failure [33]. Thus, we began an investigation into genes that could play a role in echinocandin adaptation or tolerance. Disruption of the RNA-binding protein, Ssd1, yielded minor increases in echinocandin susceptibility (Table 1), as others have also noted [31]. To determine this factor’s influence on echinocandin resistance, we disrupted the *SSD1* gene in both *FKS1* and *FKS2* resistant mutants. We discovered a reversal in echinocandin resistance following disruption of *SSD1*. Interestingly, this reversal was exclusive to *FKS1* mutants; *SSD1* disruption did not alter the echinocandin susceptibilities of *FKS2* mutants (Table 1). The reversal of resistance observed in *FKS1* mutants was complemented through expression of *SSD1* from plasmid (Table 2). Furthermore, disruption of *SSD1* from either wild type or *FKS* mutant strains led to FK506 hyper-susceptibility, similar to the *FKS1* knockout strain (Table 1).

**Figure 4:**
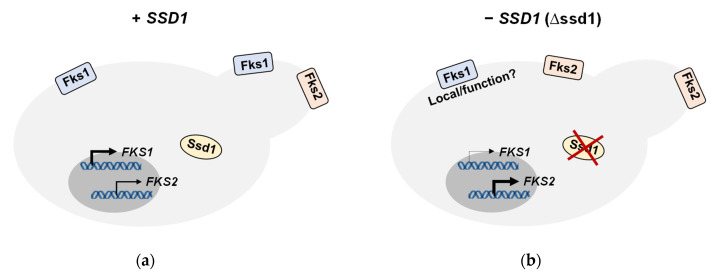
Model displaying the effects on *FKS1* and *FKS2* gene and protein with (**a**) and without (**b**) *SSD1*. These effects are more pronounced within *FKS* mutants. The Fks1 protein in cells without *SSD1* may or may not reach the plasma membrane and/or be functional. Relative, not actual, amount of Fks1/2 protein displayed; model not drawn to scale.

From these data, *SSD1* appeared to play a role in *FKS1* and/or *FKS2* regulation leading to the observed differential phenotype. Our subsequent gene and protein expression studies demonstrated a trending decrease in *FKS1* gene and protein and increase in *FKS2* gene and protein following *SSD1* disruption, particularly within the *FKS* mutant strains (Figure 2 and Figure 3). These data also suggest that *SSD1* is partially controlling *FKS1* expression, and in turn, leading to decreases in Fks1 protein and compensatory increases in *FKS2* gene and protein expression (Figure 4). However, due to the limited decrease in *FKS1* gene and protein expression, it is possible to postulate that *SSD1* also contributes to the proper transportation of Fks1 protein to the cell membrane and/or its functionality. This would help to explain the near complete reversal in Fks1-mediated echinocandin resistance and near complete sensitivity to FK506. Since Ssd1 is an RNA-binding protein and exerts post-transcriptional control of gene transcripts, we predict that Ssd1 is controlling *FKS1* indirectly. In fact, neither *FKS1* nor *FKS2* were identified in *S. cerevisiae* screens for Ssd1 bound mRNAs [18,19]; although, these studies were not performed in the presence of an echinocandin. While it is possible that Ssd1 does bind *FKS1* and/or *FKS2* in *C. glabrata*, we would expect the *FKS1* transcript levels to be either unchanged or even increased after *SSD1* disruption if Ssd1 were directly responsible for *FKS1* transcript delivery to the proper cellular location.

Because *SSD1* is responsible for delivering cell wall related genes to areas of growth or remodeling, it is understandable that loss of *SSD1* in *S. cerevisiae* has been reported to lead to weakened cell walls and activation of the cell wall integrity (CWI) pathway [34,35]. As stated in the introduction, CWI pathway activation leads to increases in *FKS2* expression, which is also dependent upon calcineurin. Although these studies have been performed in *S. cerevisiae*, our data support a similar activation of *FKS2* following loss of *SSD1* in *C. glabrata*. The increased expression of *FKS2* triggered by the CWI pathway is probably due to decreased levels of Fks1, in addition to decreases in other cell wall-related proteins (Figure 4). How exactly *SSD1* is leading to decreased *FKS1* expression and/or Fks1 functionality is to be determined. It is likely responsible for proper expression of a regulator (or regulators) that is essential for *FKS1* expression, and possibly, localization and/or function.

As previously mentioned, treating *C. glabrata FKS2* mutants with a calcineurin inhibitor, such as the immune modulator tacrolimus (FK506), will reverse their acquired echinocandin resistance [7]. Here, we demonstrated reversal of *FKS1*-meditaed resistance upon deletion of *SSD1*. Further investigation into this mechanism is warranted to 1) better understand mechanistic regulation of *FKS1* and *FKS2* and 2) uncover inhibitors of the *SSD1* related pathway as possible treatment options of refractory *C. glabrata* infections.

## Figures and Tables

**Table 1:** Disruption of *SSD1* selectively reverses *FKS1*-mediated echinocandin resistance and induces FK506 sensitivity. *SSD1* was disrupted in wild type and equivalent *FKS1* and *FKS2* mutants and caspofungin (CSF), micafungin (MCF), and FK506 minimum inhibitory concentrations (MICs) determined. Results are representative of three independent experiments.

24 h MICs (µg/mL)				
	Strain	CSF	MCF	FK506
	wild type	0.03	0.03	32
	∆ssd1	0.016	0.008	≤0.5
	∆fks1	0.008	0.016	≤0.5
	∆fks2	0.016	0.03	32
Fks1-				
	625delF	2	1	16
	625delF ∆ssd1	0.03	0.008	≤0.5
	S629P	2	1	32
	S629P ∆ssd1	0.25	0.03	1
Fks2-				
	659delF	2	1	32
	659delF ∆ssd1	2	0.5	≤0.5
	S663P	2	1	64
	S663P ∆ssd1	2	1	2

**Table 2:** Restoration of echinocandin resistance in *fks1 ∆ssd1* mutants upon heterologous expression of *SSD1*. The *SSD1* coding region was cloned onto pCN-PDC1 via gap-repair (pSSD1). Results are representative of three independent experiments. CSF, caspofungin; MCF, micafungin.

24 h MICs (µg/mL)		
Strain	CSF	MCF
WT + empty	0.016	0.016
WT + pSSD1	0.016	0.016
∆ssd1 + empty	0.016	0.008
625delF + empty	4	1
625delF + pSSD1	4	1
625delF ∆ssd1 + empty	0.12	0.016
625delF ∆ssd1 + pSSD1	4	0.5
S629P + empty	4	2
S629P + pSSD1	2	2
S629P ∆ssd1 + empty	0.06	0.016
S629P ∆ssd1 + pSSD1	2	2

## Supplementary Materials

The following are available online at https://www.mdpi.com/2309-608X/6/3/143/s1, Table S1: Primers used in this study.

## References

[1] Castanheira M., Messer S.A., Jones R.N., Farrell D.J., Pfaller M.A. (2014). Activity of echinocandins and triazoles against a contemporary (2012) worldwide collection of yeast and moulds collected from invasive infections. Int. J. Antimicrob. Agents.

[2] Alexander B.D., Johnson M.D., Pfeiffer C.D., Jimenez-Ortigosa C., Catania J., Booker R., Castanheira M., Messer S.A., Perlin D.S., Pfaller M.A. (2013). Increasing echinocandin resistance in Candida glabrata: Clinical failure correlates with presence of FKS mutations and elevated minimum inhibitory concentrations. Clin. Infect. Dis..

[3] Lamoth F., Lockhart S.R., Berkow E.L., Calandra T. (2018). Changes in the epidemiological landscape of invasive candidiasis. J. Antimicrob. Chemother..

[4] Pappas P.G., Kauffman C.A., Andes D.R., Clancy C.J., Marr K.A., Ostrosky-Zeichner L., Reboli A.C., Schuster M.G., Vazquez J.A., Walsh T.J. (2016). Clinical Practice Guideline for the Management of Candidiasis: 2016 Update by the Infectious Diseases Society of America. Clin. Infect. Dis..

[5] Perlin D.S. (2015). Mechanisms of echinocandin antifungal drug resistance. Ann. N. Y. Acad. Sci..

[6] Mazur P., Morin N., Baginsky W., el-Sherbeini M., Clemas J.A., Nielsen J.B., Foor F. (1995). Differential expression and function of two homologous subunits of yeast 1,3-beta-D-glucan synthase. Mol. Cell. Biol..

[7] Katiyar S.K., Alastruey-Izquierdo A., Healey K.R., Johnson M.E., Perlin D.S., Edlind T.D. (2012). Fks1 and Fks2 are functionally redundant but differentially regulated in Candida glabrata: Implications for echinocandin resistance. Antimicrob. Agents Chemother..

[8] Gomar-Alba M., Morcillo-Parra M.A., Olmo M.L. (2015). Response of yeast cells to high glucose involves molecular and physiological differences when compared to other osmostress conditions. FEMS Yeast Res..

[9] Zhao C., Jung U.S., Garrett-Engele P., Roe T., Cyert M.S., Levin D.E. (1998). Temperature-induced expression of yeast FKS2 is under the dual control of protein kinase C and calcineurin. Mol. Cell. Biol.

[10] Brisse S., Pannier C., Angoulvant A., de Meeus T., Diancourt L., Faure O., Muller H., Peman J., Viviani M.A., Grillot R. (2009). Uneven distribution of mating types among genotypes of Candida glabrata isolates from clinical samples. Eukaryot. Cell.

[11] Muller H., Hennequin C., Gallaud J., Dujon B., Fairhead C. (2008). The asexual yeast Candida glabrata maintains distinct a and alpha haploid mating types. Eukaryot. Cell.

[12] Gabaldon T., Fairhead C. (2019). Genomes shed light on the secret life of Candida glabrata: Not so asexual, not so commensal. Curr. Genet..

[13] Garcia-Effron G., Katiyar S.K., Park S., Edlind T.D., Perlin D.S. (2008). A naturally occurring proline-to-alanine amino acid change in Fks1p in Candida parapsilosis, Candida orthopsilosis, and Candida metapsilosis accounts for reduced echinocandin susceptibility. Antimicrob. Agents Chemother..

[14] Garcia-Effron G., Lee S., Park S., Cleary J.D., Perlin D.S. (2009). Effect of Candida glabrata FKS1 and FKS2 mutations on echinocandin sensitivity and kinetics of 1,3-beta-D-glucan synthase: Implication for the existing susceptibility breakpoint. Antimicrob. Agents Chemother..

[15] Niimi K., Woods M.A., Maki K., Nakayama H., Hatakenaka K., Chibana H., Ikeda F., Ueno K., Niimi M., Cannon R.D. (2012). Reconstitution of high-level micafungin resistance detected in a clinical isolate of Candida glabrata identifies functional homozygosity in glucan synthase gene expression. J. Antimicrob. Chemother..

[16] Wu J., Chen X., Cai L., Tang L., Liu L. (2015). Transcription factors Asg1p and Hal9p regulate pH homeostasis in Candida glabrata. Front. Microbiol..

[17] Bernardo R.T., Cunha D.V., Wang C., Pereira L., Silva S., Salazar S.B., Schroder M.S., Okamoto M., Takahashi-Nakaguchi A., Chibana H. (2017). The CgHaa1-Regulon Mediates Response and Tolerance to Acetic Acid Stress in the Human Pathogen Candida glabrata. G3 (Bethesda).

[18] Hogan D.J., Riordan D.P., Gerber A.P., Herschlag D., Brown P.O. (2008). Diverse RNA-binding proteins interact with functionally related sets of RNAs, suggesting an extensive regulatory system. PLoS Biol..

[19] Jansen J.M., Wanless A.G., Seidel C.W., Weiss E.L. (2009). Cbk1 regulation of the RNA-binding protein Ssd1 integrates cell fate with translational control. Curr. Biol..

[20] Kurischko C., Kuravi V.K., Herbert C.J., Luca F.C. (2011). Nucleocytoplasmic shuttling of Ssd1 defines the destiny of its bound mRNAs. Mol. Microbiol..

[21] Kurischko C., Kim H.K., Kuravi V.K., Pratzka J., Luca F.C. (2011). The yeast Cbk1 kinase regulates mRNA localization via the mRNA-binding protein Ssd1. J. Cell Biol.

[22] Zordan R.E., Ren Y., Pan S.J., Rotondo G., De Las Penas A., Iluore J., Cormack B.P. (2013). Expression plasmids for use in Candida glabrata. G3 (Bethesda).

[23] Healey K.R., Katiyar S.K., Raj S., Edlind T.D. (2012). CRS-MIS in Candida glabrata: Sphingolipids modulate echinocandin-Fks interaction. Mol. Microbiol..

[24] Johnson M.E., Edlind T.D. (2012). Topological and mutational analysis of Saccharomyces cerevisiae Fks1. Eukaryot. Cell.

[25] National Committee for Clinical Laboratory Standards (2017). Reference Method for Broth Dilution ANTIFUNGAL Susceptibility Testing of Yeasts. Clinical and Laboratory Standards Institute Document M27.

[26] Healey K.R., Nagasaki Y., Zimmerman M., Kordalewska M., Park S., Zhao Y., Perlin D.S. (2017). The Gastrointestinal Tract Is a Major Source of Echinocandin Drug Resistance in a Murine Model of Candida glabrata Colonization and Systemic Dissemination. Antimicrob. Agents Chemother..

[27] Livak K.J., Schmittgen T.D. (2001). Analysis of relative gene expression data using real-time quantitative PCR and the 2(-Delta Delta C(T)) Method. Methods.

[28] Li Q.Q., Skinner J., Bennett J.E. (2012). Evaluation of reference genes for real-time quantitative PCR studies in Candida glabrata following azole treatment. BMC Mol. Biol..

[29] Park S., Kelly R., Kahn J.N., Robles J., Hsu M.J., Register E., Li W., Vyas V., Fan H., Abruzzo G. (2005). Specific substitutions in the echinocandin target Fks1p account for reduced susceptibility of rare laboratory and clinical Candida sp. isolates. Antimicrob. Agents Chemother..

[30] Hou X., Healey K.R., Shor E., Kordalewska M., Ortigosa C.J., Paderu P., Xiao M., Wang H., Zhao Y., Lin L.Y. (2019). Novel FKS1 and FKS2 modifications in a high-level echinocandin resistant clinical isolate of Candida glabrata. Emerg. Microbes Infect..

[31] Schwarzmuller T., Ma B., Hiller E., Istel F., Tscherner M., Brunke S., Ames L., Firon A., Green B., Cabral V. (2014). Systematic phenotyping of a large-scale Candida glabrata deletion collection reveals novel antifungal tolerance genes. PLoS Pathog..

[32] Healey K.R., Perlin D.S. (2018). Fungal Resistance to Echinocandins and the MDR Phenomenon in Candida glabrata. J. Fungi.

[33] Shields R.K., Nguyen M.H., Press E.G., Kwa A.L., Cheng S., Du C., Clancy C.J. (2012). The presence of an FKS mutation rather than MIC is an independent risk factor for failure of echinocandin therapy among patients with invasive candidiasis due to Candida glabrata. Antimicrob. Agents Chemother..

[34] Kaeberlein M., Guarente L. (2002). Saccharomyces cerevisiae MPT5 and SSD1 function in parallel pathways to promote cell wall integrity. Genetics.

[35] Arias P., Diez-Muniz S., Garcia R., Nombela C., Rodriguez-Pena J.M., Arroyo J. (2011). Genome-wide survey of yeast mutations leading to activation of the yeast cell integrity MAPK pathway: Novel insights into diverse MAPK outcomes. BMC Genom..

