# Supplementary material for: Differential Regulation of Echinocandin Targets Fks1 and Fks2 in Candida glabrata by the Post-Transcriptional Regulator Ssd1
Source: J Fungi (Basel). 2020 Aug 21;6(3):143. doi: 10.3390/jof6030143 (PMC7558938; doi:10.3390/jof6030143)
Supplement: Supplementary file 1 [file jof-06-00143-s001.pdf]

Journal of Fungi

Supplementary Materials: Differential Regulation of Echinocandin Targets Fks1 and Fks2 in *Candida glabrata* by the Post-Transcriptional Regulator Ssd1

Table S1. Primers used in this study.

Primer†	Application	Sequence (5′-3′)‡
CgFKS1c1757F	hotspot 1 PCR	ACGTCGCTTCTCAAACCTTC
CgFKS1c2225R	hotspot 1 PCR/sequence	GCGTTCCAGACTTGGGAAAT
CgFKS1c1674F	screen/sequence	GTTGCAGTCGCTACATTGCTA
CgFKS1c3918F	hotspot 2 PCR	CGCTCTTGCACACGAATCTA
CgFKS1c4225R	hotspot 2 PCR/sequence	CACCACCAACAGTCAAATCG
CgFKS2c1790F	hotspot 1 PCR	CGATTATGCCATTAGGTGGTC
CgFKS2c2165R	hotspot 1 PCR/sequence	CCAACAGAGAAGACAGTGTTGA
CgFKS2c1419F	screen/sequence	GGATTATGCACGTTTCCGTC
CgFKS2c3930F	hotspot 2 PCR	GCATCCTGGTTTCCATTTGA
CgFKS2c4312R	hotspot 2 PCR/sequence	GATTGGATCAGACGTTATACATTG
CgSSD1-TRP1F	ssd1Δ	CCTTTTCCCCAGAACAAAGGACAGAAGTGTAAGCGCCGAG GGTCACTATTCTAACTGAAAACCCCAAATGTCTGTTATTAAT TTCACAGG
CgSSD1-TRP1R	ssd1Δ	TATTATAATTACTCAAAAAAGCAACAACGTAAAACTTACAA GGTTCATAGTAATTTAAGGAATGAGCCTATTTCTTAGCATTT TTGACGA
CgSSDu280F	screen/sequence	GTCCCTTGTTGTACAGGTG
ScTRP1c417R	screen	CGAATGAGGTTTCTGTGAAGC
CgSSD1c583R	screen	AGTGCGTTCTGGACCTGTTG
CgSSD1u143F	screen	TTCAGCGTCTTCCCTATCGC
CgSSD1u172F	PCR/screen	CTTTGCGCCATTCGTTCTCC
CgSSD1d301R	PCR	AACAAGCAGTGAAATGAATGTC
pCN-PDC1-SSD 1F	gap-repair	CAAAAAACATTAACATCTAGAACTAGTGGATCCCCCGGGCT GCAGGAATTCATGTCGAAGTTTCATCGCCA
pCN-PDC1-SSD 1R	gap-repair	TGGTGGTAGCTGTGGGTTGTGTTCTCGAGGTCGACGGTATCG ATAAGCTTTTAGTGTTTTGGTTCCACTTTC
pCN-PDC1F	PCR/screen	GAGACCAGACTAATACAACTG
pCN-reverse	PCR/sequence	GTTGCCTGCTACGTAAAGTG
CgSSD1c894R	sequence	GTTTGAGTTGCTTCTGCGATG
CgSSD1c1739R	sequence	GGACGTTGTTTCAATGAGCC
CgSSD1c2476R	sequence	CACCTGACGATCTCTTTCTG
CgSSD1c3280R	sequence	CATCTGAATAAGGAACATCG
CgFKS1expF	qRT-PCR	CAATTGGCAGAACACCGATCCCAA
CgFKS1expR	qRT-PCR	AGTTGGGTTGTCCGTACTCATCGT
CgFKS2expF	qRT-PCR	TACCAACCAGAAGACCAACAGAATGG
CgFKS2expR	qRT-PCR	TCACCACCGCTGATGTTTGGGT
CgRDN5.8F	qRT-PCR	CTTGGTTCTCGCATCGATGA
CgRDN5.8R	qRT-PCR	GGCGCAATGTGCGTTCA

†Numbers in primer names correspond to nucleotide location upstream (u) or within the coding region (c) relative to the start codon, or downstream (d) relative to the stop codon. ‡Underlined regions of deletion primers correspond to *S. cerevisiae TRP1* coding sequences; underlined regions of gap-repair cloning primers correspond to *C. glabrata SSD1* upstream or downstream sequences, and non-underlined regions correspond to sequences on pCN-PDC1 surrounding EcoRV restriction site.
